# Effect of medication adherence on long-term all-cause-mortality and hospitalization for cardiovascular disease in 65,067 newly diagnosed type 2 diabetes patients

**DOI:** 10.1038/s41598-018-30740-y

**Published:** 2018-08-15

**Authors:** Yeon-Yong Kim, Jin-Seok Lee, Hee-Jin Kang, Sang Min Park

**Affiliations:** 1 Big Data Steering Department, National Health Insurance Service, Wonju, Korea; 2 Department of Health Policy and Management, Seoul National University College of Medicine, Seoul, Korea; 3 Institute of Health Policy and Management, Seoul National University Research Center, Seoul, Korea; 4 Department of Family Medicine, Seoul National University College of Medicine, Seoul, Korea; 5 Department of Biomedical Sciences, Seoul National University College of Medicine, Seoul, Korea

## Abstract

This study determined the effects of anti-diabetic medication adherence on the long-term all-cause mortality and hospitalization for cerebrovascular disease and myocardial infarction among newly diagnosed patients. The study used retrospective cohort from the National Health Insurance Service. Study participants were 65,076 newly diagnosed type 2 diabetes mellitus patients aged ≥40 years. The medication adherence was evaluated from the proportion of days covered (PDC) between 2006 and 2007. Outcome variables were mortality, newly diagnosed cerebrovascular disease (CVD) and myocardial infarction (MI) in 2008–2017. Cox-proportional hazard regression analysis was performed. After adjusting for sex, age, monthly contribution, insurance type, medical institution type, Charlson comorbidity index score, disability, hypertension, and active ingredients of oral hypoglycemic agents, the adjusted hazard ratio (aHR) for all-cause-mortality of the lowest PDC group (<0.20) was 1.45 (95% confidence interval [CI] = 1.36–1.54) as compared to the highest PDC (≥0.8). The aHR for all-cause-mortality associated with PDC levels of 0.60–0.79, 0.40–0.59, and 0.20–0.39 were 1.19, 1.26, and 1.34, respectively (*P*_trend_ < 0.001). Compared to the highest PDC group, diabetic patients with the lowest PDC had elevated risk for CVD (aHR = 1.41; 95% CI = 1.30–1.52; *P*_trend_ < 0.001). Improving anti-diabetic medication adherence among newly diagnosed type 2 diabetes mellitus patients is essential to the reduce risk for cardiovascular disease and long-term all-cause mortality.

## Introduction

Continuity of care, a provider factor, and medication adherence, a patient factor, in chronic disease management affect both the health outcome and the healthcare expenditure in chronic disease management^1–3^. Continuous treatment in diabetes mellitus (DM) is particularly important for preventing diabetes-related complications^4^. Among the various factors associated with lifestyle modification, such as dietary control and exercise, medication adherence is the key determinant for diabetes management^5^. Several studies have shown a correlation between medication adherence and intermediate outcomes, such as blood glucose, glycosylated hemoglobin level, and healthcare utilization among DM patients^6–12^. However, only a few studies focused on the association between mortality, ultimate health outcome, and medication nonadherence^13–16^. These studies focused solely on poor medication adherence and failed to address the effects of the degree of adherence in patient’s health, which is more important than poor adherence alone to establish a policy basis for intervention. The limitations of these studies also include short follow-up periods and insufficient sample sizes. Furthermore, most studies examining intermediate and ultimate health outcome do not focus on newly diagnosed patients. These patients are less affected by the past treatment history, which is a potentially confounding variable. By focusing on newly diagnosed patients, it is feasible to identify the un-confounded effects of medication adherence on mortality. The significance of initial treatment and health outcomes of newly diagnosed patients are important, but to our knowledge, they have not been studied sufficiently. This study determined the effects of medication adherence on cardiovascular disease-related hospitalization and long-term all-cause mortality among newly diagnosed type 2 diabetic patients.

## Methods

### Data source

We used customized retrospective cohort data from the National Health Information Database (NHID) of the National Health Insurance Service (NHIS-2016-1-058). The NHID includes healthcare utilization data based on claims data, that include the entire population of South Korea; and eligibility data containing socioeconomic status, such as insurance type and monthly contributions based on the income level. More than 99% of claims data, including prescription records required for calculating medication adherence, were submitted electronically. Because of the separation of prescription and dispensing systems in South Korea, almost all diabetic medications require prescription. Using National Health Insurance claim data in South Korea started in 1986, 892 domestic and international research papers have been identified until 2015^17^. We used the NHID collected from 2002 to 2017 in this study.

### Study participants and variables

Study participants were newly diagnosed type 2 DM patients aged ≥40 years in 2006. We assessed medication adherence in 2006–2007, then followed up with data on hospitalization and death starting from 2008 to 2017. For reference, the prevalence of type 2 diabetes in South Korea was 12.6% with 6.6% newly diagnosed patients in 2001–2002 based on a community-based cohort study^18^.

In our study, newly diagnosed patients were defined as those who did not visit healthcare institutions in 2002–2004 for type 2 DM (with or without complications), but did visit in 2005 for type 2 DM according to the International Classification of Disease (ICD) 10th revision codes (E11). The analysis did not include those patients with cancer (C00-D48), myocardial infarction (MI) (I21-I23), and cerebrovascular disease (CVD) (I60-I69, G45-G46) until 2005. We used a whole diagnosis codes from the healthcare utilization data to meet the exclusion criteria and a primary diagnosis code to meet the inclusion criteria. In order to evaluate the level of comorbidities, the Charlson Comorbidity Index (CCI) was calculated from the entire inpatient and outpatient healthcare utilization records (only primary diagnosis code) from 2002–2004. We used the same scoring criteria that used in a study by Steffen *et al*. for comorbidities^19^. Socioeconomic status variables, such as age, sex, disability, insurance type, and monthly contributions in 2006, were used as independent variables from the eligibility data section of the NHID.

The medication adherence of newly diagnosed patients was evaluated, using the healthcare utilization database section of the NHID between 2006 and 2007. The proportion of days covered (PDC), the recently preferred method of measuring medication adherence^20^, was used as an indicator of medication adherence. During a uniform period of 2 years to obtain accurate long-term adherence information, the PDC was calculated from the patients’ prescription record, which contains the information on the duration of a prescription (sum of total prescription days) of oral hypoglycemic agents^21,22^. Participants without at least two outpatient visits, which are essential for calculating the PDC, and who were hospitalized for more than 90 days during the intervention period (2006–2007) which reflects a serious condition and is influenced mainly by the healthcare provider, were excluded from the analysis in order to focus on the PDC assessment. The information of active ingredient of oral hypoglycemic agent was classified into three groups according to the main ingredient type (more than half of the whole prescription days) (biguanide, sulfonylurea, and others) based on the anatomical therapeutic chemical (ATC) classification system of the World Health Organization Collaboration Centre for Drug Statistics Methodology. Due to consider the effect of the antihypertensive medication, the whole diagnosis codes (I10-I15) and prescription of antihypertensive medication during 2005–2007 were used to determine whether hypertension was treated. The information of the main medical institution type (primary, secondary, and tertiary level institution), the PDC, and active ingredients of oral hypoglycemic agents were collected from the healthcare utilization data for the intervention period (2006–2007).

Outcome variables were mortality (from the eligibility database of the NHID) and newly diagnosed CVD and MI (based on the ICD-10 code from the healthcare utilization database of the NHID). To identify the incident (new-onset) cases, we used the first hospitalization for CVD or MI as an operational definition of a new onset diagnosis. The date of death and date of first admission under the ICD-10 codes for CVD and MI defined as the date of the event. Data on participants were examined from the index date of follow-up (January 1, 2008) until December 31, 2017. A total of 65,067 participants were included in the study after excluding the entire missing value and the cases which exclusion conditions were met.

### Statistical methods

The survival analysis was performed using the cox-proportional hazard regression model. The model was adjusted for age, sex, disability, insurance type, monthly contributions, medical institution type, the CCI, hypertension, and active ingredients of oral hypoglycemic agents. We also performed stratified analysis by independent variables, with PDC as the main independent variable of interest. All variables, including PDC were used for analysis as categorical variables, not continuous variables. PDC was categorized into five groups (<0.2, 0.2–0.39, 0.4–0.59, 0.6–0.79, 0.8-). The date of death, CVD onset, and MI onset were the outcome variables, and considered as continuous variables. The proportional hazards assumption in the Cox model was assessed with graphical methods. The Wald chi-square test was conducted for evaluating linear trends in the hazard ratio of the regression. The sensitivity analyses for additional variables of lifestyle factor, such as smoking, alcohol drinking frequency in a week, obesity (body mass index more than 30 kg/m^2^), systolic and diastolic blood pressure, and fasting blood glucose were performed using those cases who participated a national health screening programme in 2006–2007 (N = 28,158). The SAS Enterprise Guide 7.1 program was used.

### Ethics approval

This study received an exemption from the Korea National Institute for Bioethics Policy Institutional Review Board (P01–201607–21–001).

## Results

### General characteristics of study participants

General characteristics of the study participants based on the PDC are shown in Table 1. The majority of participants were men in their 50 s who paid contributions of Korean Won (KRW) 20,000–39,999 (~US$ 18–37) monthly. They were dependents of the employees who were insured and generally visited the clinic, had a zero CCI score. They were not disabled, and were comprised ≥0.80 of the PDC proportion (34.2% of the study participants). They were not patients of hypertension, and predominantly prescribed sulfonylurea.

**Table 1:** General characteristics of newly-diagnosed diabetic patients (study participants) according to the proportion of days covered (PDC) (*N* = *65*,*067*, *% in column)*.

	N	Proportion of days covered (PDC) (2006–2007)									
		≥0.80		0.60–0.79		0.40–0.59		0.20–0.39		<0.20	
		N	%	N	%	N	%	N	%	N	%
Total (% in row)	65,067	22,261	34.2	11,459	17.6	10,279	15.8	10,393	16.0	10,675	16.4
**Sex**											
Men	39,808	13,227	59.4	7,014	61.2	6,416	62.4	6,474	62.3	6,677	62.6
Women	25,259	9,034	40.6	4,445	38.8	3,863	37.6	3,919	37.7	3,998	37.5
**Age**											
40–49	18,752	5,634	25.3	3,555	31.0	3,079	30.0	3,213	30.9	3,271	30.6
50–59	22,173	7,844	35.2	3,934	34.3	3,558	34.6	3,429	33.0	3,408	31.9
60–69	16,524	6,191	27.8	2,766	24.1	2,503	24.4	2,478	23.8	2,586	24.2
70+	7,618	2,592	11.6	1,204	10.5	1,139	11.1	1,273	12.3	1,410	13.2
**Insurance type**											
The self-employed	21,042	6,552	29.4	3,791	33.1	3,482	33.9	3,506	33.7	3,711	34.8
Families of the self-employed	8,713	2,990	13.4	1,551	13.5	1,379	13.4	1,406	13.5	1,387	13.0
The employ insured	13,586	4,734	21.3	2,453	21.4	2,081	20.3	2,160	20.8	2,158	20.2
Dependents of the employ insured	21,726	7,985	35.9	3,664	32.0	3,337	32.5	3,321	32.0	3,419	32.0
**Monthly contribution (KRW)**											
<20,000	9,021	2,900	13.0	1,647	14.4	1,489	14.5	1,446	13.9	1,539	14.4
20,000–39,999	15,817	5,205	23.4	2,837	24.8	2,511	24.4	2,594	25.0	2,670	25.0
40,000–59,999	12,435	4,231	19.0	2,151	18.8	2,003	19.5	1,989	19.1	2,061	19.3
60,000–79,999	10,326	3,593	16.1	1,819	15.9	1,577	15.3	1,615	15.5	1,722	16.1
80,000–99,999	6,848	2,447	11.0	1,194	10.4	1,022	9.9	1,050	10.1	1,135	10.6
100,000+	10,620	3,885	17.5	1,811	15.8	1,677	16.3	1,699	16.4	1,548	14.5
**Charlson Comorbidity Index (2002–2004)**											
0	49,634	17,325	77.8	8,790	76.7	7,790	75.8	7,803	75.1	7,926	74.3
1	12,199	3,916	17.6	2,139	18.7	1,959	19.1	2,035	19.6	2,150	20.1
2+	3,234	1,020	4.6	530	4.6	530	5.2	555	5.3	599	5.6
**Disability**											
No	61,218	21,029	94.5	10,820	94.4	9,631	93.7	9,781	94.1	9,957	93.3
Yes	3,849	1,232	5.5	639	5.6	648	6.3	612	5.9	718	6.7
**Medical institution type (2006–2007)**											
Tertiary hospital	13,192	5,418	24.3	2,176	19.0	1,800	17.5	1,917	18.5	1,881	17.6
Secondary hospital	5,541	1,472	6.6	1,023	8.9	977	9.5	996	9.6	1,073	10.1
Clinic	41,209	13,275	59.6	7,417	64.7	6,847	66.6	6,729	64.8	6,941	65.0
Public health center	5,125	2,096	9.4	843	7.4	655	6.4	751	7.2	780	7.3
**Main active ingredient of oral hypoglycemic agent (2006–2007)**											
Biguanide	14,605	5,189	23.3	2,502	21.8	2,166	21.1	2,297	22.1	2,451	23.0
Sulfonylurea	28,625	9,324	41.9	5,011	43.7	4,716	45.9	4,684	45.1	4,890	45.8
Others	21,837	7,748	34.8	3,946	34.4	3,397	33.1	3,412	32.8	3,334	31.2
**Hypertension (2005–2007)**											
No	33,264	11,357	51.0	6,243	54.5	5,207	50.7	5,041	48.5	5,416	50.7
Yes	31,803	10,904	49.0	5,216	45.5	5,072	49.3	5,352	51.5	5,259	49.3
**Main outcome (2008–2017)**											
Death	9,161	2,728	12.3	1,513	13.2	1,484	14.4	1,617	15.6	1,819	17.0
Cerebrovascular disease	5,286	1,584	7.1	906	7.9	829	8.1	948	9.1	1,019	9.6
Myocardial infarction	1,294	426	1.9	229	2.0	199	1.9	237	2.3	203	1.9

*The age, sex, disability, insurance type, and monthly contributions are taken from 2006.

### Results of the Cox proportional hazard regression analysis

**Figure 1:**
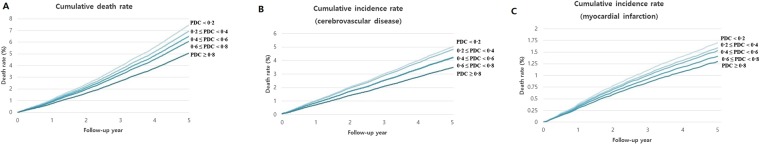
Five year age and sex standardized cumulative death (**A**) and incidence rate of cerebrovascular disease (**B**) and myocardial infarction (**C**) according to the proportion of the days covered (PDC). *Age and sex standardized with 2005 Korean Census population.

The survival analysis was performed using a Cox proportional hazard regression model. The adjusted hazard ratio (aHR) of the lowest PDC for all-cause mortality was 1.45 (95% confidence interval [CI] = 1.36–1.54) as compared to the highest PDC (≥0.80), after adjusting for factors, including sex, age, monthly contribution, insurance type, medical institution type, CCI score, disability, hypertension, and active ingredients of oral hypoglycemic agents (Table 2). The HR for all-cause mortality based on the PDC levels of 0.60–0.79, 0.40–0.59, and 0.20–0.39 were 1.19, 1.26, and 1.34, respectively (p for trend < 0.001). The HR of the non-adjusted model (model 1) was higher than that of the multivariate-adjusted model (model 2) in the lowest PDC group. The effects of the lowest PDC on all-cause mortality appeared to be greater in men than in women based on the stratified analysis, using sex as the stratification variable (Table 2). The 5-year age and sex standardized cumulative mortality rates of the lowest and highest PDC groups were 7.46% and 5.06%, respectively (P < 0.001). The 5-year age and sex standardized cumulative incidence of CVD and MI in the lowest and highest PDC groups were 5.00% and 3.48%, and 0.86% and 0.83%, respectively (Fig. 1).

**Table 2:** Cox-proportional hazard regression models for the all-cause death by the proportion of days covered (PDC) with stratification by sex.

	Proportion of days covered (PDC)									P for trend
	≥0.80	0.60–0.79		0.40–0.59		0.20–0.39		<0.20		
		Hazard ratio	95% CI	Hazard ratio	95% CI	Hazard ratio	95% CI	Hazard ratio	95% CI	
Total No. of participants	N = 22,261	N = 11,459		N = 10,279		N = 10,393		N = 10,675		
Total person-years	210,743.9	107,681.9		96,106.9		96,505.9		98,115.7		
All [No. of death]	N = 2,728	N = 1,513		N = 1,484		N = 1,617		N = 1,819		
Model 1	Reference	1.20	1.13–1.28	1.29	1.21–1.37	1.37	1.29–1.46	1.47	1.39–1.57	<0.001
Model 2		1.19	1.12–1.27	1.26	1.18–1.34	1.34	1.26–1.43	1.45	1.36–1.54	<0.001
Men [No. of death]	N = 1,776	N = 1,009		N = 998		N = 1,050		N = 1,0.170		
Model 1	Reference	1.24	1.14–1.33	1.33	1.23–1.44	1.39	1.29–1.50	1.50	1.39–1.62	<0.001
Model 2		1.22	1.13–1.32	1.30	1.20–1.40	1.35	1.25–1.46	1.46	1.35–1.57	<0.001
Women [No. of death]	N = 952	N = 504		N = 486		N = 567		N = 649		
Model 1	Reference	1.12	1.01–1.25	1.18	1.05–1.31	1.31	1.18–1.45	1.39	1.26–1.53	<0.001
Model 2		1.12	1.00–1.25	1.15	1.03–1.28	1.28	1.15–1.42	1.37	1.24–1.51	<0.001

Model 1: adjusted for age and sex (only age in stratified analysis). Model 2: adjusted for age, sex, disability, CCI, monthly contribution, insurance type, medical institution type, hypertension, and active ingredients of oral hypoglycemic agents (except sex in stratified analysis).

The results of Cox-proportional hazard regression analysis for first admission, related to CVD and MI, are presented in Table 3. Compared to the highest PDC group (≥0.80), diabetic patients with the lowest PDC (<0.20) had an elevated risk for CVD-related first admission (aHR = 1.41; 95% CI = 1.30–1.52; *P*_trend_ < 0.001). However, there was no statistically significant increase in the risk of MI (aHR = 1.02; 95% CI = 0.86–1.21; *P*_trend_ = 0.138). Stratified analysis, using sex, age, monthly contribution, CCI, disability, hypertension, and main active ingredient of oral hypoglycemic agent showed gradual increases of HR associated with decreased PDC for all-cause mortality (Supplementary Table 1) and CVD hospitalization (Supplementary Table 2). Such trends were revealed in all stratified subgroups for all-cause mortality. The highest HR for all-cause mortality was 1.89 (95% CI = 1.56–2.28; *P*_trend_ < 0.001) in patients aged 40–49 years. In the sensitivity analyses for adjusting lifestyle factors (N = 28,158) (Supplementary Table 3), the HR of the lowest PDC group was the highest, and significance of P for trend was similar with the main results (Table 3).

**Table 3:** Cox-proportional hazard regression for the cerebrovascular disease (CVD) and myocardial infarction (MI) by the proportion of days covered (PDC) with the stratification by sex.

	Proportion of days covered (PDC)									P for trend
	≥0.80	0.60–0.79		0.40–0.59		0.20–0.39		<0.20		
		Hazard ratio	95% CI	Hazard ratio	95% CI	Hazard ratio	95% CI	Hazard ratio	95% CI	
Total No. of participants	N = 22,261	N = 11,459		N = 10,279		N = 10,393		N = 10,675		
**Cerebrovascular disease**										
Total person-years	204,354.8	104,059.3		92,813.8		92,862.4		94,075.1		
All [No. of event]	N = 1,584	N = 906		N = 829		N = 948		N = 1,019		
Model 1	Reference	1.21	1.11–1.31	1.21	1.12–1.32	1.38	1.27–1.50	1.44	1.33–1.56	<0.001
Model 2		1.19	1.10–1.29	1.18	1.09–1.29	1.35	1.24–1.46	1.41	1.30–1.52	<0.001
Men [No. of event]	N = 931	N = 558		N = 501		N = 577		N = 594		
Model 1	Reference	1.26	1.14–1.40	1.23	1.10–1.37	1.43	1.29–1.59	1.43	1.29–1.59	<0.001
Model 2		1.24	1.12–1.38	1.19	1.07–1.33	1.39	1.25–1.54	1.39	1.26–1.55	<0.001
Women [No. of event]	N = 653	N = 348		N = 328		N = 371		N = 425		
Model 1	Reference	1.12	0.99–1.28	1.18	1.04–1.35	1.31	1.15–1.48	1.44	1.27–1.63	<0.001
Model 2		1.12	0.98–1.27	1.16	1.01–1.32	1.27	1.12–1.45	1.41	1.25–1.59	<0.001
**Myocardial infarction**										
Total person-years	209,197.3	106,730.9		95,272.1		95,506.6		97,376.5		
All [No. of event]	N = 426	N = 229		N = 199		N = 237		N = 203		
Model 1	Reference	1.10	0.94–1.29	1.05	0.89–1.24	1.25	1.06–1.46	1.04	0.88–1.23	0.094
Model 2		1.09	0.93–1.28	1.03	0.87–1.22	1.23	1.05–1.44	1.02	0.86–1.21	0.138
Men [No. of event]	N = 326	N = 168		N = 150		N = 172		N = 150		
Model 1	Reference	1.03	0.86–1.24	1.01	0.83–1.22	1.16	0.96–1.39	0.98	0.81–1.20	0.566
Model 2		1.02	0.85–1.23	0.99	0.81–1.20	1.14	0.95–1.37	0.97	0.80–1.18	0.595
Women [No. of event]	N = 100	N = 61		N = 49		N = 65		N = 53		
Model 1	Reference	1.29	0.94–1.77	1.15	0.81–1.61	1.48	1.08–2.02	1.14	0.81–1.59	0.159
Model 2		1.28	0.93–1.76	1.11	0.79–1.57	1.42	1.03–1.94	1.10	0.79–1.54	0.236

Model 1: adjusted for age and sex (only age in stratified analysis). Model 2: adjusted for age, sex, disability, CCI, monthly contribution, insurance type, medical institution type, hypertension, and active ingredients of oral hypoglycemic agents (except sex in stratified analysis).

## Discussions

In this large cohort study with a long duration follow-up, we found that patients with poor medication adherence within the first 2 years following DM diagnosis had an increased risk for long-term all-cause mortality and cardiovascular disease. Specifically, we found that poor medication adherence increased the mortality rate by 45%, and the incidence of CVD by 41%.

Dose-response associations were observed between the level of medication adherence and risk of death and cardiovascular disease. The gradual increase in mortality and morbidity associated with the reduction in the level of PDC (except MI) was identified, using a trend test. Although several previous studies^13–16^ have demonstrated an association between medication adherence and mortality in DM patients, they did not assess the dose-response association between medication adherence and mortality or morbidity. Policy interventions to improve medication adherence to a maximum level may reduce the 5-year cumulative mortality rate from 7.46% to 5.06% among patients with poor medication adherence. Consequently, it can prevent 240 deaths per 10,000 individuals. Similarly, it would also prevent 152 incident cases of CVD per 10,000 individuals.

We used medication adherence instead of intermediate outcomes, such as glycated hemoglobin level and lifestyle modification. The intermediate outcome is influenced by both patient and physician factors. In terms of the patient, it is possible that patients with poor medication adherence have an irregular lifestyle and indifference to healthy behavior, which is known as the “health adherer effect”^23^. They may be less sensitive to health information and visit healthcare facilities less frequently than the “adherers.” In terms of the healthcare provider, the glycated hemoglobin test may be restrictive because of equipment problems or the physician’s discretion. The results of this study suggest that medication adherence is more important than the intermediate outcomes. Maintaining good medication adherence can be achieved via education and management programs for both physicians and patients^24^. Only 34.2% of the newly diagnosed patients in the current study had good medication adherence, which is higher than the 0.80 PDC level reported in previous studies^25^. The rate of good medication adherence was lower than the rate found in previous studies that did not focus on newly diagnosed patients^15,26^. In research of medication of hypertensive patients, the percentage of newly diagnosed patients with good medication adherence ranged between 8 and 29%^27–29^, which was lower than that previously observed good medication adherence among hypertensive patients (72%)^30^. Thus, the difference in adherence levels between those reported in previous studies and the ones observed in our study is probably due to the difference between patients with a newly diagnosed status and those with a previously diagnosed status.

We have focused on medication adherence within newly diagnosed DM patients for several reasons. First, to the best of our knowledge, only a few previous studies assessed these associations in patients with newly diagnosed DM. Previous studies generally included populations that were previously treated or management program-registered diabetic patients, and therefore, failed to address the importance of improving anti-diabetic medication adherence among newly diagnosed patients. Second, it is known that newly diagnosed DM patients already have diabetes-related complications. Iraj *et al*.^31^ reported that patients newly diagnosed with DM have neuropathy (24–52%) and hypertension (23–59%). Good medication adherence in newly diagnosed patients makes it possible to control these complications. The initial treatment of newly diagnosed DM patients has been known to be essential to prevent these complications^32^. Asymptomatic diabetic patients are also at risk for coronary artery disease^33^; thus, treatment, including both lifestyle modification and medication, should not be delayed in these patients. Third, the mortality risk is higher in patients during the pre-diabetes stage than in patients with adequate blood glucose control^34^. Therefore, it is helpful to control glucose levels in newly diagnosed diabetic patients. It is possible to expand this result to treatment guidelines and policy intervention. If the insurer, especially under the single insurer system such as the one used in South Korea, and policy makers monitor medication adherence performance in the early-stage of DM, and then control the payment system based on the performance level, providers will be more likely to effectively manage newly diagnosed patients. This is especially important in younger patients, as evidenced by the highest HR in patients aged 40–49 years in our study. It can reduce the mortality rate of the young patients by 89% and minimize the burden of premature death caused by poor medication adherence.

There are some limitations to this study. First, unmeasured factors affecting medication adherence, such as social support, satisfaction with care, patient knowledge, and physician characteristics^35,36^ were not analyzed. Because the available variables in the NHID were limited, the HbA1c, kidney function, lipid profiles, and blood pressure level were not included in the main analysis, although these factors can affect the health outcomes. However, sensitivity analysis for adjusting smoking, alcohol drinking, obesity, systolic and diastolic blood pressure, and fasting blood glucose was also performed, and the results were similar regardless of various lifestyle factors. Second, we used PDC as a proxy indicator of medication adherence. This was calculated from the prescription records; hence, real medication consumption and timing may not accurately match the PDC level. Third, the analysis was performed, using claims data and diagnosis codes, and the actual use of medication can be influenced by the payment system. However, an operational definition was established to overcome this issue.

In conclusion, we found that poorer medication adherence led to the worsening of health outcomes, which was not addressed in the previous studies. Improving patient’s adherence to anti-diabetic medication in the early stage, following the diagnosis of DM is necessary to prevent the risk of long-term all-cause mortality and cardiovascular disease.

## Electronic supplementary material

Supplementary tables

## Data Availability

The datasets analysed during the current study are not publicly available due to the policy of the National Health Insurance Service in Korea.
