# Supplementary material for: Effect of medication adherence on long-term all-cause-mortality and hospitalization for cardiovascular disease in 65,067 newly diagnosed type 2 diabetes patients
Source: Sci Rep. 2018 Aug 15;8:12190. doi: 10.1038/s41598-018-30740-y (PMC6093904; doi:10.1038/s41598-018-30740-y)
Supplement: Supplementary file 1 — Supplementary tables [file 41598_2018_30740_MOESM1_ESM.doc]

**Supplementary information**

**Effect of medication adherence on long-term all-cause-mortality and hospitalization for cardiovascular disease in 65,067 newly diagnosed type 2 diabetes patients**

Yeon-Yong Kim^1^, Jin-Seok Lee^2,3^, Hee-Jin Kang^1^, Sang Min Park*^4, 5^

*^1^ Big Data Steering Department, National Health Insurance Service, Wonju, Korea*

*^2^ Department of Health Policy and Management, Seoul National University College of Medicine, Seoul, Korea*

*^3^ Institute of Health Policy and Management, Seoul National University Research Center, Seoul, Korea*

*^4^ Department of Family Medicine, Seoul National University College of Medicine, Seoul, Korea*

*^5^ Department of Biomedical Sciences, Seoul National University College of Medicine, Seoul, Korea*

Yeon-Yong Kim [*yong115@daum.net*](mailto:yong115@daum.net) *26464 32 Geongang-ro, Wonju-si, Gangwon-do, Korea*

Jin-Seok Lee [*phealth@snu.ac.kr*](mailto:phealth@snu.ac.kr) *03080 101 Daehak-ro, Jongno-gu, Seoul, Korea*

Hee-Jin Kang [*mini2016@nhis.or.kr*](mailto:mini2016@nhis.or.kr) *26464 32 Geongang-ro, Wonju-si, Gangwon-do, Korea*

Sang Min Park [*smpark.snuh@gmail.com*](mailto:smpark.snuh@gmail.com) *03080 101 Daehak-ro, Jongno-gu, Seoul, Korea*

*Corresponding author: Sang Min Park, MD, PhD

Department of Family Medicine & Department of Biomedical Science, Seoul National University, College of Medicine

03080 101 Daehak-ro, Jongno-gu, Seoul, Korea

Email: smpark.snuh@gmail.com, Phone: 82-2-2072-3331, Fax: 82-2-766-3276

Key words: Medication adherence, Mortality, Oral Antidiabetics, Chronic Care Management, Cardiovascular Complications

Running title: Medication adherence and mortality in newly diagnosed Type 2 DM

Supplementary Table 1. Stratified cox-proportional hazard regression for the all-cause death by the proportion of days covered (PDC)

|  | Proportion of days covered (PDC) | | | | | | | |  |  | P for trend |
| --- | --- | --- | --- | --- | --- | --- | --- | --- | --- | --- | --- |
|  | ≥0.80 | | 0.60-0.79 | | 0.40-0.59 | | 0.20-0.39 | | <0.20 | |  |
| **Stratification variable** |  |  | Hazard ratio | 95% CI | Hazard ratio | 95% CI | Hazard ratio | 95% CI | Hazard ratio | 95% CI |  |
| **Death [No. of event]** | N=2,728 | | N=1,513 | | N=1,484 | | N=1,617 | | N=1,819 | |  |
| **Age** |  | |  |  |  |  |  |  |  |  |  |
| 40-49 | Reference | | 1.15 | 0.93-1.42 | 1.44 | 1.18-1.77 | 1.70 | 1.40-2.07 | 1.89 | 1.56-2.28 | <0.001 |
| 50-59 |  |  | 1.13 | 0.98-1.30 | 1.22 | 1.06-1.41 | 1.26 | 1.10-1.45 | 1.53 | 1.35-1.75 | <0.001 |
| 60-69 |  |  | 1.19 | 1.07-1.33 | 1.23 | 1.10-1.38 | 1.28 | 1.14-1.43 | 1.37 | 1.23-1.52 | <0.001 |
| 70+ |  |  | 1.23 | 1.11-1.36 | 1.23 | 1.11-1.37 | 1.32 | 1.19-1.45 | 1.32 | 1.20-1.46 | <0.001 |
| **Monthly contribution (KRW)** |  | |  |  |  |  |  |  |  |  |  |
| <40,000 | Reference | | 1.22 | 1.11-1.35 | 1.30 | 1.17-1.43 | 1.39 | 1.26-1.53 | 1.53 | 1.39-1.68 | <0.001 |
| 40,000-79,999 |  |  | 1.16 | 1.04-1.29 | 1.28 | 1.15-1.42 | 1.39 | 1.25-1.54 | 1.46 | 1.32-1.62 | <0.001 |
| 80,000+ |  |  | 1.19 | 1.05-1.35 | 1.18 | 1.04-1.34 | 1.20 | 1.06-1.35 | 1.31 | 1.16-1.48 | <0.001 |
| **Charlson comorbidity index** |  | |  |  |  |  |  |  |  |  |  |
| 0 | Reference | | 1.21 | 1.13-1.30 | 1.25 | 1.16-1.35 | 1.36 | 1.27-1.47 | 1.48 | 1.38-1.58 | <0.001 |
| 1 |  |  | 1.19 | 1.04-1.37 | 1.28 | 1.12-1.47 | 1.23 | 1.07-1.42 | 1.42 | 1.24-1.61 | <0.001 |
| 2+ |  |  | 0.89 | 0.68-1.17 | 1.18 | 0.91-1.52 | 1.40 | 1.10-1.77 | 1.22 | 0.97-1.54 | 0.012 |
| **Disability** |  | |  |  |  |  |  |  |  |  |  |
| No | Reference | | 1.17 | 1.10-1.25 | 1.25 | 1.17-1.34 | 1.34 | 1.25-1.43 | 1.44 | 1.35-1.53 | <0.001 |
| Yes |  |  | 1.36 | 1.09-1.70 | 1.34 | 1.07-1.67 | 1.38 | 1.11-1.71 | 1.54 | 1.25-1.88 | 0.001 |
| **Hypertension** |  | |  |  |  |  |  |  |  |  |  |
| No | Reference | | 1.19 | 1.07-1.31 | 1.33 | 1.20-1.47 | 1.41 | 1.27-1.56 | 1.48 | 1.34-1.63 | <0.001 |
| Yes |  |  | 1.19 | 1.10-1.29 | 1.21 | 1.12-1.32 | 1.29 | 1.19-1.40 | 1.42 | 1.32-1.53 | <0.001 |
| **Main active ingredient of oral hypoglycemic agent** |  | |  |  |  |  |  |  |  |  |  |
| Biguanide | Reference | | 1.12 | 0.96-1.31 | 1.28 | 1.10-1.50 | 1.20 | 1.02-1.40 | 1.41 | 1.22-1.63 | <0.001 |
| Sulfonylurea |  |  | 1.21 | 1.10-1.33 | 1.19 | 1.08-1.30 | 1.35 | 1.23-1.47 | 1.39 | 1.27-1.52 | <0.001 |
| Others |  |  | 1.19 | 1.07-1.32 | 1.34 | 1.21-1.49 | 1.40 | 1.26-1.55 | 1.54 | 1.39-1.70 | <0.001 |

Stratified analysis was adjusted for sex and all other variables in table 1.

Supplementary Table 2. Stratified cox-proportional hazard regression for the CVD and MI by the proportion of days covered (PDC)

|  | Proportion of days covered (PDC) | | | | | | | |  |  | P for trend |
| --- | --- | --- | --- | --- | --- | --- | --- | --- | --- | --- | --- |
|  | ≥0.80 | | 0.60-0.79 | | 0.40-0.59 | | 0.20-0.39 | | <0.20 | |  |
| **Stratification variable** |  |  | Hazard ratio | 95% CI | Hazard ratio | 95% CI | Hazard ratio | 95% CI | Hazard ratio | 95% CI |  |
| **Cerebrovascular disease [No. of event]** | N=1,584 | | N=906 | | N=829 | | N=948 | | N=1,019 | |  |
| **Age** |  | |  |  |  |  |  |  |  |  |  |
| 40-49 | Reference | | 1.38 | 1.10-1.75 | 1.55 | 1.22-1.96 | 1.65 | 1.31-2.07 | 1.98 | 1.59-2.47 | <0.001 |
| 50-59 |  |  | 1.14 | 0.97-1.33 | 1.11 | 0.95-1.30 | 1.45 | 1.25-1.68 | 1.42 | 1.22-1.65 | <0.001 |
| 60-69 |  |  | 1.20 | 1.05-1.38 | 1.18 | 1.02-1.36 | 1.25 | 1.09-1.44 | 1.31 | 1.15-1.51 | 0.001 |
| 70+ |  |  | 1.19 | 1.00-1.41 | 1.11 | 0.93-1.32 | 1.23 | 1.04-1.45 | 1.27 | 1.08-1.49 | 0.028 |
| **Monthly contribution (KRW)** |  | |  |  |  |  |  |  |  |  |  |
| <40,000 | Reference | | 1.20 | 1.06-1.36 | 1.13 | 0.99-1.29 | 1.41 | 1.24-1.59 | 1.37 | 1.21-1.56 | <0.001 |
| 40,000-79,999 |  |  | 1.16 | 1.00-1.33 | 1.17 | 1.02-1.35 | 1.29 | 1.12-1.48 | 1.50 | 1.32-1.71 | <0.001 |
| 80,000+ |  |  | 1.24 | 1.05-1.46 | 1.27 | 1.08-1.50 | 1.31 | 1.11-1.54 | 1.31 | 1.11-1.55 | 0.002 |
| **Charlson comorbidity index** |  | |  |  |  |  |  |  |  |  |  |
| 0 | Reference | | 1.19 | 1.09-1.31 | 1.15 | 1.04-1.27 | 1.33 | 1.21-1.46 | 1.41 | 1.29-1.55 | <0.001 |
| 1 |  |  | 1.21 | 1.00-1.46 | 1.34 | 1.12-1.61 | 1.33 | 1.11-1.60 | 1.30 | 1.08-1.56 | 0.005 |
| 2+ |  |  | 1.06 | 0.72-1.56 | 1.03 | 0.69-1.52 | 1.61 | 1.14-2.26 | 1.68 | 1.21-2.33 | 0.003 |
| **Disability** |  | |  |  |  |  |  |  |  |  |  |
| No | Reference | | 1.22 | 1.12-1.32 | 1.18 | 1.08-1.29 | 1.36 | 1.25-1.48 | 1.43 | 1.32-1.55 | <0.001 |
| Yes |  |  | 0.93 | 0.67-1.28 | 1.21 | 0.91-1.61 | 1.19 | 0.89-1.59 | 1.18 | 0.89-1.56 | 0.388 |
| **Hypertension** |  | |  |  |  |  |  |  |  |  |  |
| No | Reference | | 1.13 | 1.00-1.29 | 1.19 | 1.04-1.36 | 1.28 | 1.12-1.46 | 1.39 | 1.22-1.57 | <0.001 |
| Yes |  |  | 1.24 | 1.11-1.38 | 1.18 | 1.05-1.31 | 1.39 | 1.25-1.54 | 1.42 | 1.28-1.57 | <0.001 |
| **Main active ingredient of oral hypoglycemic agent** |  | |  |  |  |  |  |  |  |  |  |
| Biguanide | Reference | | 1.21 | 1.00-1.47 | 1.25 | 1.03-1.53 | 1.15 | 0.95-1.41 | 1.58 | 1.32-1.89 | <0.001 |
| Sulfonylurea |  |  | 1.17 | 1.03-1.32 | 1.10 | 0.97-1.24 | 1.33 | 1.18-1.49 | 1.31 | 1.17-1.48 | <0.001 |
| Others |  |  | 1.22 | 1.06-1.40 | 1.26 | 1.09-1.45 | 1.46 | 1.28-1.67 | 1.43 | 1.25-1.64 | <0.001 |
| **Myocardial infarction [No. of event]** | N=426 | | N=229 | | N=199 | | N=237 | | N=203 | |  |
| **Age** |  | |  |  |  |  |  |  |  |  |  |
| 40-49 | Reference | | 1.74 | 1.19-2.56 | 1.67 | 1.12-2.50 | 1.87 | 1.27-2.76 | 1.81 | 1.23-2.68 | 0.010 |
| 50-59 |  |  | 1.04 | 0.79-1.37 | 0.92 | 0.69-1.23 | 1.13 | 0.85-1.48 | 1.01 | 0.75-1.35 | 0.825 |
| 60-69 |  |  | 0.98 | 0.73-1.32 | 0.88 | 0.64-1.22 | 1.07 | 0.79-1.45 | 0.85 | 0.61-1.17 | 0.709 |
| 70+ |  |  | 0.89 | 0.59-1.33 | 1.03 | 0.69-1.52 | 1.17 | 0.81-1.69 | 0.76 | 0.50-1.14 | 0.412 |
| **Monthly contribution (KRW)** |  | |  |  |  |  |  |  |  |  |  |
| <40,000 | Reference | | 1.09 | 0.84-1.43 | 1.04 | 0.79-1.37 | 1.32 | 1.02-1.71 | 1.06 | 0.81-1.40 | 0.311 |
| 40,000-79,999 |  |  | 1.09 | 0.83-1.42 | 0.98 | 0.74-1.30 | 1.16 | 0.89-1.52 | 0.96 | 0.73-1.28 | 0.718 |
| 80,000+ |  |  | 1.09 | 0.80-1.49 | 1.10 | 0.80-1.52 | 1.17 | 0.85-1.60 | 1.07 | 0.77-1.47 | 0.901 |
| **Charlson comorbidity index** |  | |  |  |  |  |  |  |  |  |  |
| 0 | Reference | | 1.09 | 0.90-1.31 | 1.14 | 0.94-1.38 | 1.30 | 1.08-1.56 | 1.07 | 0.88-1.30 | 0.086 |
| 1 |  |  | 1.12 | 0.79-1.59 | 0.78 | 0.52-1.16 | 0.95 | 0.65-1.37 | 0.89 | 0.61-1.30 | 0.546 |
| 2+ |  |  | 0.94 | 0.44-2.02 | 0.48 | 0.18-1.28 | 1.38 | 0.70-2.70 | 0.88 | 0.42-1.85 | 0.356 |
| **Disability** |  | |  |  |  |  |  |  |  |  |  |
| No | Reference | | 1.10 | 0.93-1.29 | 1.04 | 0.87-1.24 | 1.23 | 1.04-1.45 | 1.04 | 0.87-1.24 | 0.176 |
| Yes |  |  | 0.98 | 0.52-1.82 | 0.95 | 0.50-1.81 | 1.18 | 0.65-2.15 | 0.85 | 0.45-1.61 | 0.928 |
| **Hypertension** |  | |  |  |  |  |  |  |  |  |  |
| No | Reference | | 1.05 | 0.82-1.33 | 1.13 | 0.88-1.45 | 1.15 | 0.90-1.49 | 1.03 | 0.80-1.33 | 0.794 |
| Yes |  |  | 1.12 | 0.90-1.39 | 0.95 | 0.75-1.20 | 1.26 | 1.02-1.55 | 1.00 | 0.80-1.25 | 0.126 |
| **Main active ingredient of oral hypoglycemic agent** |  | |  |  |  |  |  |  |  |  |  |
| Biguanide | Reference | | 1.51 | 1.06-2.15 | 1.24 | 0.84-1.83 | 1.02 | 0.68-1.54 | 1.63 | 1.15-2.31 | 0.031 |
| Sulfonylurea |  |  | 1.01 | 0.78-1.29 | 1.00 | 0.78-1.29 | 1.40 | 1.12-1.76 | 0.83 | 0.63-1.08 | 0.003 |
| Others |  |  | 1.00 | 0.77-1.31 | 0.98 | 0.74-1.30 | 1.09 | 0.83-1.43 | 1.01 | 0.76-1.34 | 0.974 |

Stratified analysis was adjusted for sex and all other variables in table 1.

Supplementary Table 3. Sensitivity analysis for adjusting lifestyle factors of the subjects who participated health screening programme in 2006-2007 (N=28,158)

|  | Proportion of days covered (PDC) | | | | | | | |  |  | P for trend |
| --- | --- | --- | --- | --- | --- | --- | --- | --- | --- | --- | --- |
|  | ≥0.80 | | 0.60-0.79 | | 0.40-0.59 | | 0.20-0.39 | | <0.20 | |  |
|  |  |  | Hazard ratio | 95% CI | Hazard ratio | 95% CI | Hazard ratio | 95% CI | Hazard ratio | 95% CI |  |
| **Death** [No. of event] | N=981 | | N=532 | | N=551 | | N=536 | | N=652 | |  |
| **Total** | Reference | | 1.14 | 1.03-1.27 | 1.31 | 1.18-1.45 | 1.25 | 1.13-1.39 | 1.37 | 1.24-1.52 | <0.001 |
| Men |  |  | 1.17 | 1.04-1.33 | 1.36 | 1.20-1.54 | 1.23 | 1.08-1.40 | 1.36 | 1.20-1.53 | <0.001 |
| Women |  |  | 1.07 | 0.88-1.31 | 1.18 | 0.97-1.44 | 1.29 | 1.07-1.57 | 1.40 | 1.17-1.68 | 0.003 |
| **Cerebrovascular disease** [No. of event] | N=669 | | N=378 | | N=336 | | N=361 | | N=410 | |  |
| **Total** | Reference | | 1.17 | 1.03-1.33 | 1.14 | 1.00-1.30 | 1.23 | 1.08-1.40 | 1.28 | 1.13-1.45 | 0.001 |
| Men |  |  | 1.21 | 1.03-1.41 | 1.09 | 0.92-1.29 | 1.26 | 1.07-1.47 | 1.26 | 1.08-1.48 | 0.011 |
| Women |  |  | 1.12 | 0.91-1.39 | 1.24 | 1.00-1.53 | 1.19 | 0.96-1.48 | 1.31 | 1.07-1.61 | 0.094 |
| **Myocardial infarction** [No. of event] | N=173 | | N=105 | | N=81 | | N=82 | | N=85 | |  |
| **Total** | Reference | | 1.24 | 0.97-1.58 | 1.05 | 0.80-1.37 | 1.06 | 0.81-1.38 | 1.03 | 0.79-1.33 | 0.537 |
| Men |  |  | 1.20 | 0.91-1.58 | 0.96 | 0.70-1.31 | 1.04 | 0.77-1.40 | 1.05 | 0.78-1.41 | 0.712 |
| Women |  |  | 1.42 | 0.85-2.37 | 1.34 | 0.79-2.27 | 1.13 | 0.65-1.97 | 0.92 | 0.51-1.64 | 0.521 |

Adjusted for age, sex, disability, CCI, monthly contribution, smoking, alcohol drinking frequency in a week, obesity (body mass index 30 kg/m^2^ or over), insurance type, medical institution type, hypertension, active ingredients of oral hypoglycemic agents, systolic and diastolic blood pressure, and fasting blood glucose (except sex in stratified analysis)
